# Quality of life and clinical outcomes in rectal cancer patients treated on a 1.5T MR-Linac within the MOMENTUM study^☆^

**DOI:** 10.1016/j.ctro.2023.100721

**Published:** 2024-01-04

**Authors:** L.A. Daamen, J.M. Westerhoff, A.M. Couwenberg, P.M. Braam, H. Rütten, M.D. den Hartogh, J.P. Christodouleas, W.A. Hall, H.M. Verkooijen, M.P.W. Intven

**Affiliations:** a University Medical Center Utrecht, Division of Imaging and Oncology, Utrecht, The Netherlands; b The Netherlands Cancer Institute, Department of Radiation Oncology, Amsterdam, The Netherlands; c Radboud University Medical Center, Department of Radiation Oncology, Nijmegen, The Netherlands; d Radiotherapiegroep, The Netherlands; e Elekta AB, Stockholm, Sweden; f Hospital of the University of Pennsylvania, Department of Radiation Oncology, Philadelphia, PA, United States; g Medical College of Wisconsin, Department of Radiation Oncology, Milwaukee, WI, United States; h University Medical Center Utrecht, Department of Radiation Oncology, Utrecht, The Netherlands

**Keywords:** MR-guided radiotherapy, Rectal cancer, Short-course radiation therapy, Quality of Life, Toxicity, Clinical outcomes, Patient-reported outcomes, Response assessment

## Abstract

**Background and purpose:**

This study assessed quality of life (QoL) and clinical outcomes in rectal cancer patients treated with magnetic resonance (MR) guided short-course radiation therapy (SCRT) on a 1.5 Tesla (T) MR-Linac during the first 12 months after treatment.

**Materials and methods:**

Rectal cancer patients treated with 25 Gy SCRT in five fractions with curative intent in the Netherlands (2019–2022) were identified in MOMENTUM (NCT04075305). Toxicity (CTCAE v5) and QoL (EORTC QLQ-C30 and -CR29) was primarily analyzed in patients without metastatic disease (M0) and no other therapies after SCRT. Patients who underwent tumor resection were censored from surgery. A generalized linear mixed-model was used to investigate clinically meaningful (≥10) and significant (P < 0.05) QoL changes. Clinical and pathological complete response (cCR and pCR) rates were calculated in patients in whom response was documented.

**Results:**

A total of 172 patients were included, of whom 112 patients were primarily analyzed. Acute and late radiation-induced high-grade toxicity were reported in one patient, respectively. CCR was observed in 8/64 patients (13 %), 14/37 patients (38 %) and 13/16 patients (91 %) at three, six and twelve months; pCR was observed in 3/69 (4 %) patients. After 12 months, diarrhea (mean difference [MD] −17.4 [95 % confidence interval [CI] −31.2 to −3.7]), blood and mucus in stool (MD −31.1 [95 % CI −46.4 to −15.8]), and anxiety (MD –22.4 [95 % CI −34.0 to −10.9]) were improved.

**Conclusion:**

High-field MR-guided SCRT for the treatment of patients with rectal cancer is associated with improved disease-related symptom management and functioning one year after treatment.

## Highlights

• High-grade radiation-induced toxicity was reported in only two patients.
• Treatment resulted in improved disease-related symptom management and functioning.
• Treatment was, however, also associated with worsened impotence.
• Other quality of life domains remained stable or stabilized after six to 12 months.
• This study supports investigation of dose escalation or boosting with MR-guided RT.

## Introduction

Rectal cancer is typically treated with multimodal therapy, including surgery, systemic therapy and radiation therapy (RT). For patients with intermediate risk rectal tumors, defined as cT3c-dN0M0 or cT1-3N1M0 mesorectal fascia clear (MRF-), the recommended treatment strategy in Northern Europe is short-course radiotherapy (SCRT; 25 Gray (Gy) in five fractions) followed by (delayed) tumor resection [1], [2], [3]. SCRT is also used in patients not eligible for tumor resection to provide palliation of symptoms, in elderly not eligible for chemoradiation, in combination with chemotherapy to treat locally advanced rectal cancer (LARC), for early tumors to allow organ-preservation, and in combination with chemotherapy, immunotherapy and metastasectomy for the treatment of metastatic rectal cancer [1], [2], [3], [4], [5].

Although neoadjuvant SCRT has not been associated with survival benefits, it reduces the risk of locoregional recurrence or tumor progression [6], [7]. In addition, a prolonged interval between SCRT and surgery has been found to improve tumor downstaging [8], [9]. Sufficient downstaging of the tumor could allow for organ-preservation in half of rectal cancer patients, as demonstrated in studies investigating other total neoadjuvant therapy regimens [10], [11]. However, after SCRT with delay, radiation-induced toxicity has also been reported, mainly consisting of bowel dysfunction, and requiring hospital admission in 7 % of patients [12], [13]. With a rising number of patients eligible for a watch-and-wait approach, this is becoming increasingly relevant. To reduce RT-related toxicity without compromising treatment efficacy, the 1.5 Tesla (T) Magnetic Resonance Linear Accelerator (MR-Linac) holds great promise for the treatment of rectal cancer [14], [15], [16]. This technology uses online adaptive MR-guided RT, which provides superior soft tissue visualization and daily plan adaptation based on the actual anatomy. MR-guided RT makes it possible to reduce the radiation field for rectal tumors by 1/3rd as compared with conventional techniques, which may result in less RT-related toxicity and a better quality of life (QoL) [17], [18], [19].

Only few single-center studies have been conducted on the use of the 1.5T MR-Linac to treat rectal cancer patients. The ‘Multi-OutcoMe EvaluatioN of radiation Therapy Using the MR-Linac (MOMENTUM) registry provides the unique opportunity to assess clinical and patient-reported outcomes (PROs) in a multi-institutional patient cohort [20]. Therefore, this study aims to assess QoL and clinical outcomes, including toxicity, disease control and survival during the first 12 months after MR-guided RT in rectal cancer patients treated with SCRT on a 1.5T MR-Linac in the MOMENTUM study. These results can serve as a benchmark and support the design of both comparative effectiveness studies and novel treatment studies using the MR-Linac for treatment of patients with rectal cancer.

## Methods

The Strengthening the Reporting of Observational Studies in Epidemiology (STROBE) guidelines for cohort studies were followed [21].

### Study design and population

This study was performed using the MOMENTUM registry (NCT04075305). MOMENTUM is a multi-institutional, observational cohort study, in which patients treated on the 1.5T MR-Linac (Unity, Elekta) consent to the use of their pseudonymized clinical and technical patient data. In addition, patients consent to receive QoL questionnaires during follow-up after treatment [20]. For the current analysis, all patients with primary rectal cancer who received 25 Gy in five fractions with curative intent between February 2019 and April 2023 in one of four Dutch institutions (i.e., University Medical Center Utrecht, Radboud University Medical Center, Netherlands Cancer Institute, Radiotherapiegroep) were identified.

### Outcomes

Outcomes of interest were RT-related toxicity, QoL, disease control and survival, measured at three, six and 12 months after RT. Toxicity was scored according to Common Terminology Criteria for Adverse Events version 5 (CTCAE v5). Acute toxicity was defined as observed either during treatment or within the initial three months, while subsequent occurrences were categorized as late toxicity. QoL was assessed using European Organisation for Research and Treatment of Cancer Quality of Life Questionnaires (EORTC QLQ), specifically the general EORTC QLQ-C30 and colorectal cancer-specific EORTC QLQ-CR29 questionnaires [22], [23]. Oncological outcomes consisted of clinical response rate, pathological complete response (pCR) rate, disease-free interval (DFI), and overall survival (OS).

### Data collection

Patient characteristics included age, sex, and performance status (according to the Eastern Cooperative Oncology Group [ECOG] scale). Tumor characteristics comprised tumor (T), nodal (N) and metastasis (M) status and corresponding American Joint Committee on Cancer (AJCC) stage [24]. Treatment details contained therapy completion, reasons for non-completion, RT adaptation workflow, and details on other therapies at baseline and during follow-up, including type and timing of therapy. The RT adaptation workflow existed of either Adapt-to-Position (ATP), in which the dose plan is shifted to match the daily anatomy resembling rigid registration but based on MR images, or Adapt-to-Shape (ATS), in which the dose plan is adapted to the real-time shape, volume and position of the target area and organs at risk right before treatment. Follow-up data included treatment response as documented in the electronic patient file, presence and pattern of disease progression and/or recurrence after resection, vital status and survival time. Clinical response was classified as complete, partial, stable or progressive; pCR was defined as pT0N0.

### Statistical analysis

Baseline characteristics were summarized using descriptive statistics. Primarily, radiation-induced toxicity and QoL were assessed in patients without metastatic disease (M0) at baseline, excluding patients who received other therapies after RT to explicitly evaluate the effect of MR-guided SCRT. Patients who underwent resection, including Transanal Endoscopic Microsurgery (TEM), were censored from the date of surgery. High-grade toxicity (i.e., CTCAE grade 3 or higher) was reported using absolute numbers and percentages. It was assumed that if high-grade toxicity had occurred, this would have been documented in the respective patient record given its severity. Therefore, if not documented, it was assumed that high-grade toxicity had not occurred. Since this could give an underestimation of toxicity, high-grade toxicity was additionally calculated in patients in whom toxicity was prospectively scored according to CTCAE, likely providing an overestimation. Consequently, a range of values within which the true toxicity rate lies was obtained. QoL domains were calculated according to the EORTC manual [22]. High scores on functional scales represent a high level of functioning (better QoL), whilst high scores on symptom scales represent a high level of symptomatology (worse QoL). Scores were presented as mean (±standard deviation [SD]). Generalized linear mixed-model analysis was performed to assess change in QoL over time by calculating mean differences (MD) and corresponding 95 % confidence intervals (CI). Minimally important differences (MIDs) were interpreted based on Musoro et al. for available QoL domains [25]. If not specified, a ≥10 point change was considered meaningful. A sensitivity analysis was performed in the total cohort of patients with organ preservation, including patients with TEM or other additional therapies during follow-up, censoring patients from the date of surgery in case of resection. DFI and OS were assessed in the total cohort by Kaplan-Meier curves and presented as one-year probability with 95 % CI. DFI was calculated from the date of resection until local or systemic disease recurrence or last follow-up. OS was calculated from the start date of treatment until the date of death or last follow-up. Clinical response rates were obtained in non-resected patients in whom response assessment with follow-up MRI was documented; recurrence rates were determined in resected patients in whom information on recurrence was available. Oncological outcomes were stratified for disease stage according to the Dutch guidelines, i.e., low risk (cT1-3abN0M0, CRM-), intermediate risk (cT3cdN0M0 or cT1-3N1M0, CRM-), LARC (cT4, N2 or CRM+) and metastatic [1]. P-values < 0.05 were considered statistically significant.

## Results

### Study population

During the study period, 174 patients were scheduled to receive treatment on the 1.5T MR-Linac. In one patient, MR-Linac treatment was not given because of technical issues. In another patient, new pathological lymph nodes were found and the treatment schedule was changed to long course chemoradiation. Consequently, 172 patients were included: 9 patients (5 %) with low risk rectal cancer, 109 patients (63 %) with intermediate risk rectal cancer, 35 patients (21 %) with LARC and 19 patients (11 %) with metastatic disease (Table 1). Three, six and twelve months follow-up was reached by 169 patients (98 %), 163 patients (95 %), and 133 patients (77 %), respectively.

**Table 1:** Baseline characteristics of the total cohort of 172 patients primary rectal cancer treated with 5x5 Gy with curative intent on the 1.5 T MR-Linac.

	**Total** **(N = 172)**
**Age in years, median (IQR)**	61 [53–70]
**Sex, n (%)**	
Female	50 (29 %)
Male	122 (71 %)
**ECOG grade, n (%)**	
0	117 (68 %)
1	20 (12 %)
2	1 (1 %)
Missing	34 (20 %)
**Tumor status**[26]**, n (%)**	
T1	2 (1 %)
T2	36 (21 %)
T3	123 (72 %)
T4	5 (3 %)
T4a	4 (2 %)
T4b	2 (1 %)
**Lymph node status**[26]**, n (%)**	
N0	30 (17 %)
N1	114 (66 %)
N2	28 (16 %)
**Metastasis status**[26]**, n (%)**	
M0	153 (89 %)
M1	19 (11 %)
**Circumferential resection margin**	
Negative	129 (75 %)
Positive	34 (20 %)
Missing	9 (5 %)
**Risk category^1^**	
Low risk	9 (5 %)
Intermediate risk	109 (63 %)
Locally advanced	35 (21 %)
Metastasized	19 (11 %)
**Adaptation technique, n (%)**	
Adapt-To-Position (ATP)	3 (2 %)
Adapt-To-Shape (ATS)	166 (96 %)
Mixed	3 (2 %)
Induction systemic treatment*, n (%)	10 (6 %)
**Resection during follow-up**	119 (69 %)
Interval to surgery in weeks, median (IQR)	12 (2–18)
**Type of surgery**	
Low anterior resection (LAR)	68 (57 %)
Abdominoperineal Resection (APR)	26 (22 %)
Transanal Endoscopic Microsurgery (TEM)	10 (8 %)
Other	5 (4 %)
Unknown	10 (8 %)
**Other additional treatment (after RT, prior to resection)**	
Yes**	49 (28 %)
Systemic therapy	48 (28 %)
Brachytherapy	1 (1 %)
Metastasectomy	2 (1 %)
No	78 (45 %)
Unknown	45 (26 %)

Gy, Gray; T, Tesla; MR-Linac, Magnetic Resonance Linear Accelerator; IQR, interquartile range; ECOG, Eastern Cooperative Oncology Group; RT, radiotherapy.

* Systemic treatment consisted of chemotherapy and/or immunotherapy, including FOLFOX/bevacizumab, FOLFOX/panitumumab, FOLFOXIRI, CAPOX(-B), capecitabine, atezolizumab and/or bevacizumab.

** Patients could have received more than one additional therapy.

### Treatment details

One patient received one of five fractions on a conventional Linac, due to technical issues. All other patients completed treatment as planned. At baseline, ten patients (6 %) had received induction therapy (Table 1). At six months follow-up, 49/163 patients (28 %) had received additional treatment, consisting of systemic therapy in 48 patients, liver metastasectomy in two patients, and brachytherapy in one patient. During follow-up, 119 patients (69 %) were known to have had tumor resection after a median of 12 weeks (IQR 2–18 weeks), including organ-preserving TEM in ten patients (8 %). One of 133 patients who reached 12 months follow-up (6 %) had not undergone tumor resection due to distant disease progression, and 16 patients (94 %) were still under active surveillance.

Primary analysis with regard to radiation-induced toxicity and QoL was performed in 112 M0 patients with no other therapies after RT, of whom 45 patients (40 %), 26 patients (23 %), and 19 patients (17 %) reached three, six and twelve months follow-up without TEM, respectively.

### Radiation-induced toxicity

Toxicity was prospectively CTCAE graded in 26/45 patients (58 %) at three months, 5/26 patients (19 %) at six months and 6/19 patients (32 %) at twelve months. High-grade RT-related toxicity was reported in two patients. One patient (2–4 %) experienced grade 3 diarrhea within three months. Grade 4 constipation was reported in another patient (5–17 %) at 12 months of follow-up, with no documented tumor progression, followed by surgery.

### Quality of life

**Fig. 1:**
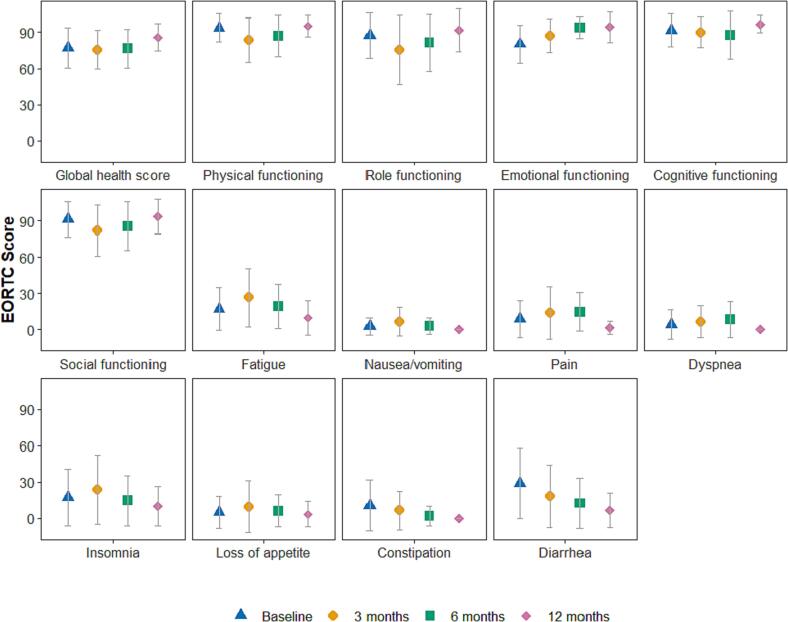
Patient reported outcomes on relevant quality of life domains of the EORTC QLQ-C30 questionnaire in M0 patients without tumor resection or other additional therapy after radiotherapy at baseline and three, six and 12 months follow-up.

At time of cohort enrollment, 96 patients (86 %) provided informed consent to receive QoL questionnaires (Appendix I & II). Treatment resulted in a decline in PRO’s during the first three to six months of follow-up in the EORTC QLQ-C30 domains of physical functioning (MD −10.1 [95 % CI −14.7 to −5.4] at three months and MD −7.2 [95 % CI −13.5 to −0.9] at six months), role functioning (MD −11.3 [95 % CI −18.9 to −3.8] at three months), social functioning (MD −9.5 [95 % CI −15.7 to −3.4] at three months), and fatigue (MD 9.5 [95 % CI 2.9 – 16.1] at three months) (Fig. 1, Table 2). Deteriorated scores returned to baseline after six months follow-up for all but the physical functioning domain. At 12 months, none of the domains showed significantly worse scores as compared with baseline. Diarrhea symptom scores showed an improvement at 12 months (MD −17.4 [95 % CI −31.2 to −3.7]), as did emotional functioning (MD 13.0 [95 % CI 5.5 – 20.6]).

**Table 2:** Mixed model analysis of patient reported outcomes on relevant domains of the EORTC QLQ-C30 questionnaire in 96 M0 patients without tumor resection or other additional therapy after radiotherapy at baseline and three, six and 12 months follow-up.

**Variable**	**Baseline**	**3 months follow-up**				**6 months follow-up**				**12 months follow-up**			
	**Mean**	**Mean**	**MD**	**LCI**	**UCI**	**Mean**	**MD**	**LCI**	**UCI**	**Mean**	**MD**	**LCI**	**UCI**
Global health score	77.2	75.1	−2.1	−7.7	3.5	76.2	−0.9	−8.5	6.6	85.7	8.5	−1.4	18.5
Physical functioning	93.7	**83.6**	**−10.1**	**−14.7**	**−5.4**	**86.6**	**−7.2**	**−13.5**	**−0.9**	90.8	−3	−11.2	5.3
Role functioning	87.5	**76.2**	**−11.3**	**−18.9**	**−3.8**	83.3	−4.2	−14.5	6	94.8	7.3	−6.2	20.8
Emotional functioning	80.3	83.5	3.2	−1.1	7.5	89	8.7	2.9	14.5	**93.3**	**13**	**5.5**	**20.6**
Cognitive functioning	91.8	89.6	−2.2	−7.3	2.8	87.8	−4	−10.9	2.8	94.3	2.4	−6.6	11.5
Social functioning	91.2	**81.7**	**−9.5**	**−15.7**	**−3.4**	87.2	−4	−12.3	4.3	92.1	0.8	−10.1	11.8
Fatigue	17	**26.5**	**9.5**	**2.9**	**16.1**	19.3	2.3	−6.6	11.3	11.8	−5.2	−17	6.5
Nausea/vomiting	2.7	6.7	4	0.5	7.5	3.8	1.1	−3.6	5.8	0.5	−2.2	−8.8	4.4
Pain	8.3	13.4	5.1	−1	11.2	14.5	6.2	−2	14.4	2.8	−5.5	−16.3	5.2
Dyspnea	4	6.3	2.3	−2.7	7.4	6.6	2.6	−4.2	9.4	0.2	−3.8	−12.9	5.2
Insomnia	17.4	23.7	6.3	−3.3	16	16	−1.4	−14.4	11.7	9.6	−7.8	−25.1	9.5
Loss of appetite	4.8	9.5	4.6	−0.9	10.1	6.3	1.5	−6	8.9	2.8	−2	−11.8	7.8
Constipation	10.6	7	−3.6	−9	1.7	5.7	−4.9	−12.2	2.3	5.6	−5	−14.5	4.4
Diarrhea	28.7	21.7	−7	−14.8	0.8	19.9	−8.7	−19.3	1.8	**11.2**	**−17.4**	**−31.2**	**−3.7**

M0, non-metastasized disease; MD, mean difference; LCI, lower confidence interval; UCI, upper confidence interval.

Bold outcomes reflect statistically significant (P < 0.05) & clinically meaningful differences according to Musoro et al^27^for available quality of life domains or according to a minimally important difference of ≥10 point change otherwise.

**Fig. 2:**
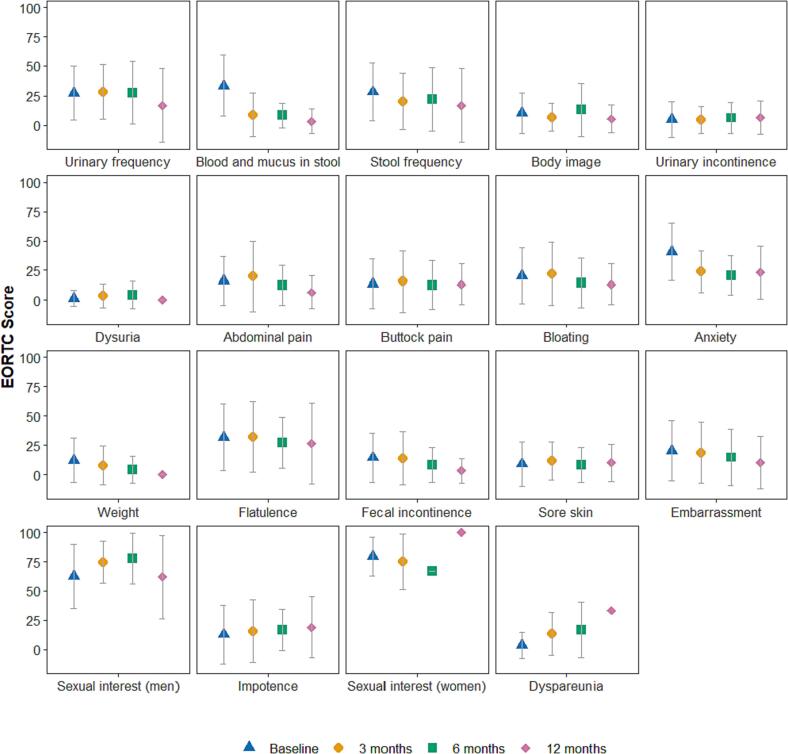
Patient reported outcomes on relevant quality of life domains of the EORTC QLQ-CR29 questionnaire in M0 patients without tumor resection or other additional therapy after radiotherapy at baseline and three, six and 12 months follow-up.

PRO’s with regard to the EORTC QLQ-CR29 domain ‘blood and mucus in stool’ showed improvement at three, six and 12 months follow-up (MD −24.8 [95 % CI –33.6 to −16.0]; MD −25.8 [95 % CI −37.5 to −14.1] and MD −31.1 [95 % CI −46.4 to −15.8], respectively) (Fig. 2, Table 3). Anxiety scores were lower at three, six and 12 months (MD −15.0 [95 % CI −21.9 to −8.1], MD −20.8 [95 % CI −29.2 to −11.0] and MD –22.4 [95 % CI −34.0 to −10.9], respectively). Other QoL domains showed no differences over time that were both clinically meaningful and statistically significant.

**Table 3:** Mixed model analysis of patient reported outcomes on relevant domains of the EORTC QLQ-CR29 questionnaire in 96 M0 patients without tumor resection or other additional therapy after radiotherapy at baseline and three, six and 12 months follow-up.

**Variable**	**Baseline**	**3 months follow-up**				**6 months follow-up**				**12 months follow-up**			
	**Mean**	**Mean**	**MD**	**LCI**	**UCI**	**Mean**	**MD**	**LCI**	**UCI**	**Mean**	**MD**	**LCI**	**UCI**
Urinary frequency	27.5	28.3	0.8	−6.8	8.4	26.1	−1.5	−12	9.1	15.6	−11.9	−24.9	1.1
Blood and mucus in stool	33.6	**8.8**	**−24.8**	**–33.6**	**−16**	**7.8**	**−25.8**	**−37.5**	**−14.1**	**2.5**	**−31.1**	**−46.4**	**−15.8**
Stool frequency	28.3	21.4	−6.9	−15.5	1.7	19.1	−9.2	−20.6	2.2	15.1	−13.1	−27.8	1.6
Body image	10.6	8.3	−2.3	−8.4	3.8	12.7	2.1	−6.1	10.3	4.9	−5.7	−16.2	4.9
Urinary incontinence	4.9	4.7	−0.2	−5.4	4.9	4.7	−0.2	−7.1	6.7	6.3	1.4	−7.5	10.3
Dysuria	1.5	3.3	1.9	−1.7	5.5	4.4	3	−1.8	7.7	0	−1.5	−7.7	4.8
Abdominal pain	16	20.9	4.9	−2.7	12.5	14.6	−1.4	−11.5	8.8	6.6	−9.4	–22.4	3.6
Anal dysfunction	13.9	16.6	2.7	−6.1	11.5	13.7	−0.3	−12.1	11.5	8.1	−5.9	−21.3	9.5
Bloating	20	22.3	2.3	−4.6	9.2	15.8	−4.2	−13.5	5	15	−5	−16.7	6.7
Anxiety	40.9	**25.8**	**−15**	**−21.9**	**−8.1**	**20.8**	**−20.1**	**−29.2**	**−11**	**18.4**	**–22.4**	**−34**	**−10.9**
Weight	11.8	8.9	−2.9	−9.6	3.7	5.5	−6.4	−15.2	2.5	0	−12.5	−24	0
Flatulence	32.1	34.7	2.6	−6.4	11.5	25.9	−6.2	−18.1	5.7	28.9	−3.2	−18.4	12
Faecal incontinence	14.7	15.1	0.3	−5.5	6.1	8.8	−5.9	−13.6	1.8	6.8	−7.9	−17.6	1.8
Sore skin	9.7	12.1	2.4	−4	8.7	6.1	−3.6	−12	4.8	2.9	−6.8	−17.6	4
Embarrassment	20.5	20.6	0.1	−7.5	7.6	14.4	−6.1	−16.1	3.9	15.6	−4.9	−17.6	7.8
Sexual interest (man)	63.2	71.5	8.3	−2.2	18.9	69.7	6.5	−7.4	20.4	57.6	−5.6	−24.5	13.2
Impotence	13.5	17.4	3.9	−4.9	12.8	18.1	4.6	−7.1	16.4	21.6	8.1	−5.9	22.2
Sexual interest (woman)	79.4	78.4	−0.9	−14.6	12.8	71.1	−8.3	−26.9	10.3	98.9	19.6	−14.3	53.5
Dyspareunia	3	14.8	11.8	−4.1	27.6	17	14	−9.4	37.3	41.1	38.1	6	70.1

M0, non-metastasized disease; MD, mean difference; LCI, lower confidence interval; UCI, upper confidence interval.

*B*old outcomes reflect statistically significant (P < 0.05) & clinically meaningful differences according to a minimally important difference of ≥ 10 point change.

Sensitivity analysis in the total cohort showed similar results (Appendix III-IV), although impotence was worse after six and 12 months (MD 10.1 [95 % CI 2 – 18.2] and MD 19 [95 % CI 9 – 28.9], respectively), as compared with baseline.

### Oncological outcomes

At three, six and 12 months, clinical complete response was observed in 8/64 patients (13 %), 14/37 patients (38 %) and 13/16 patients (81 %), respectively (Appendix V). At three months, 5/64 patients (8 %) experienced progressive disease, consisting of local progression (n = 1), distant progression (n = 2), and local and distant progression (n = 2). After six and 12 months, distant progression was seen in 1/35 patients (3 %) and 1/16 patients (6 %), respectively.

Pathological TN stage was available for 69/119 patients (58 %) after resection; pCR was observed three patients (4 %). Local recurrence after resection occurred in 1/60 resected patients (2 %) at three months and 1/52 patients (2 %) at 12 months. Distant recurrence was seen in 4/60 patients (7 %), 7/60 patients (12 %) and 11/52 patients (21 %) after three, six and 12 months, respectively. One patient (2 %) experienced both local and distant recurrence after six months follow-up.

One patient with intermediate risk rectal cancer died during 12 months follow-up, due to an unknown cause. Consequently, one-year OS was 99 % (95 % CI 97–100 %) in patients with intermediate risk rectal cancer and 100 % in patients with other disease stages.

## Discussion

This study shares unique insights from the currently largest cohort of patients who underwent high field strength MR-guided SCRT for rectal cancer. Specifically, it offers distinct follow-up details during the first year after treatment including a subset of patients who didn't have immediate resection. Acute and late radiation-induced high-grade toxicity were reported in only one patient, respectively. A clinically meaningful improvement in disease-related symptom management (i.e., less blood and mucus in stool and less diarrhea) and functioning (i.e., less anxiety) was reported. Treatment was, however, also associated with impotence in the total cohort. Other QoL domains remained stable or stabilized after six to 12 months follow-up. These findings are valuable for understanding QoL and clinical outcomes after MR-guided SCRT, which is particularly important given the growing use of watch-and-wait strategies after total neoadjuvant treatment in rectal cancer patients.

The use of RT in rectal cancer treatment has evolved over time and has become an integral component of the multidisciplinary management. SCRT is commonly used in the neoadjuvant setting for selected patients with rectal cancer, mainly for those with intermediate risk disease. Studies have demonstrated the benefit of SCRT for the reduction of local recurrence rates and improving long-term outcomes as compared to surgery alone [26], [27]. Other studies showed that SCRT results in tumor downstaging and in some cases even in a clinical or pathological complete response after delayed surgery [9], [12], [28], [29]. This has expanded the eligibility for organ-preserving treatment, aiming to maintain rectal function while effectively treating the cancer, enhancing a better QoL [30], [31]. Consequently, a prolonged interval between SCRT and surgery is increasingly recommended, although the optimal interval is still discussed [32], [33]. To adequately counsel patients about specific treatment strategies, it is crucial to study clinical outcomes. This facilitates shared-decision making and provides guidance with regard to difficult choices considering rectal cancer treatment and QoL [34].

Patients with a clinical complete response following neoadjuvant treatment can opt for a watch-and-wait approach [35]. A recent study prospectively evaluated EORTC QLQ-C30 and -CR29 scores in rectal cancer patients who were monitored using the watch-and-wait strategy during 24 months after neoadjuvant chemoradiotherapy (n = 263; 95 %) or SCRT (n = 15; 5 %) [36]. The authors concluded that patients in the watch-and-wait group experienced a good QoL with limited variation over time, with some patients reporting bowel and sexual dysfunction [36]. In line with our findings, anxiety improved after 24 months follow-up and treatment was associated with impotence. PRO’s in our cohort had greater variation over time, with a stabilization after six to 12 months. Discrepancies between these studies may be a result from a difference in patient populations, treatment strategies and time points at which outcomes were measured. The watch-and-wait study included patients with non-metastasized cancer with a clinical (near) complete response 8–12 weeks after long course chemoradiation in the fast majority of patients. In contrast, we included patients who received MR-guided SCRT, irrespective of disease stage or treatment response. Nevertheless, both studies showed a good QoL after 12 months. Particularly in the context of deteriorated patient-reported and functional outcomes after surgery, these results are promising for patients who are eligible for organ-preservation.

Another study group reported QoL of 88 patients with early-stage rectal cancer randomized to organ-preservation using TEM after SCRT (n = 27) in the TREC trial (ISRCTN14422743), and 61 older patients with a compromised performance status in the non-randomized TREC registry who were considered ineligible for total mesorectal excision [37], [38]. Similar to our findings, this study showed mild worsening of global QoL and physical functioning scores at three months after treatment, whilst scores returned to baseline values after six to 12 months. Also, a reduction in blood and mucus in stool was reported at six months post-treatment, which sustained over the remaining follow-up period, and impotence scores were found to deteriorate. The authors concluded that SCRT followed by organ-preservation is associated with good patient-reported toxicity, function and QoL; markedly better than would have been achieved with total mesorectal excision [38].

The typical SCRT regimen consists of a total radiation dose of 25 Gy divided into five daily treatments of 5 Gy each. Nevertheless, the optimal use of SCRT and its potential benefits in different patient populations continue to be studied and refined through ongoing research. Historically, SCRT has been associated with low rates of toxicity, as also demonstrated in the current study. This allows exploration of dose escalation or boosting strategies [39]. After dose escalated chemoradiotherapy in the RECTAL-BOOST trial (NCT01951521), near-complete or complete tumor regression was significantly more often observed in the intervention group (34/49 patients; 69.4 %) than in the control group (24/53 patients; 45.3 %; (OR = 2.74, 95 % CI 1.21–6.18)) [40]. Long-term outcomes showed that dose-escalated chemoradiotherapy was associated with a transient deterioration in functional and symptom scales at three and six months after treatment compared to standard chemoradiation, although scores largely recovered from 12 months onwards [41]. As compared with conventional RT techniques used in the RECTAL-BOOST trial, however, online adaptive MR-guided RT increases the precision with which irradiation doses can be delivered to the target area [17]. Therefore, it is expected that the use of MR-guided RT systems further reduces toxicity and improves patient outcomes, and enable dose escalation while preserving QoL. This is currently being investigated in the preRADAR trial [42].

The results of this study need to be interpreted in the light of several limitations. Despite that patients are prospectively registered within the MOMENTUM study, most data is collected from electronic patient files, which introduces the potential for missing data. Particularly, in a substantial part of patients, toxicity was not explicitly graded. As it was assumed that high-grade toxicity did not occur if not reported, this might give an underestimation of the true toxicity rate. Therefore, we also calculated the toxicity rate in patients in whom toxicity was prospectively scored according to CTCAE. This likely resulted in an overestimation of toxicity, thus providing a range of values within with the true toxicity lies. Calculation of clinical response and recurrence rates was limited to patients for whom this information was available, which may lead to an overestimation since patients without events are more likely to have missing data. For QoL, patients without organ-preservation where censored from the date of surgery to specifically study the effect of SCRT followed by active surveillance. A generalized mixed model analysis was performed to account for potential biases arising from missing data in QoL outcomes. Furthermore, the group of patients described in this study was quite heterogeneous. Nevertheless, presented outcomes are relevant for all patients receiving MR-guided SCRT and sensitivity analysis showed robustness of findings in more selected patient populations. The real-world outcomes of this study are therefore highly generalizable to all rectal cancer patients treated on a 1.5T MR-Linac in clinical practice. Finally, a comparison of outcomes between patients who underwent MR-guided SCRT and those treated using conventional computed tomography (CT) guided techniques would be desirable.

In conclusion, treatment of rectal cancer patients with MR-guided SCRT is associated with overall favorable QoL and clinical outcomes one year after treatment. This suggests that MR-guided SCRT is a well-tolerated local treatment option for patients with various stages of rectal cancer. Our findings support the investigation of further dose escalation or boosting strategies with MR-guided RT systems, with the aim to enhance treatment efficacy and organ-preserving treatment approaches, while maintaining a good QoL. Several such studies are ongoing and the results are eagerly anticipated (e.g., NCT04808323, NCT05916040, NCT05108428, NCT05338866) [42].

## CRediT authorship contribution statement

**JP Christodouleas:** Is an employee of Elekta. **WA Hall:** Receives institutional research and travel support from Elekta. **HM Verkooijen:** Receives research funding from Elekta.

## Declaration of competing interest

The authors declare that they have no known competing financial interests or personal relationships that could have appeared to influence the work reported in this paper.

## References

[1] Primaire behandeling rectumcarcinoom – Richtlijn – Richtlijnendatabase. https://richtlijnendatabase.nl/richtlijn/colorectaal_carcinoom_crc/primaire_behandeling_rectumcarcinoom_bij_crc.html. Published 2019. Accessed March 14, 2022.

[2] Glynne-Jones R., Wyrwicz L., Tiret E. (2018). Rectal cancer: ESMO Clinical Practice Guidelines for diagnosis, treatment and follow-up. Ann Oncol..

[3] NICE. National Institute for Health and Care Excellence. Colorectal cancer: diagnosis and management Clinical guideline. Natl Inst Heal Care Excell. 2011; (December):1-48. https://www.nice.org.uk/guidance/cg131?unlid=549491424201612410206. Accessed March 14, 2022.

[4] Bahadoer R.R., Dijkstra E.A., van Etten B. (2021). Short-course radiotherapy followed by chemotherapy before total mesorectal excision (TME) versus preoperative chemoradiotherapy, TME, and optional adjuvant chemotherapy in locally advanced rectal cancer (RAPIDO): a randomised, open-label, phase 3 trial. Lancet Oncol..

[5] Donnelly M., Ryan O.K., Ryan É.J. (2023). Total neoadjuvant therapy versus standard neoadjuvant treatment strategies for the management of locally advanced rectal cancer: network meta-analysis of randomized clinical trials [published online ahead of print, 2023 Jun 18]. Br J Surg.

[6] Chen C., Sun P., Rong J., Weng H.W., Dai Q.S., Ye S. (2015). Short course radiation in the treatment of localized rectal cancer: a systematic review and meta-analysis. Sci Rep.

[7] van Gijn W., Marijnen C.A., Nagtegaal I.D., Kranenbarg E.M., Putter H., Wiggers T. (2011). van de Velde CJ; Dutch Colorectal Cancer Group. Preoperative radiotherapy combined with total mesorectal excision for resectable rectal cancer: 12-year follow-up of the multicentre, randomized controlled TME trial. Lancet Oncol.

[8] Erlandsson J., Fuentes S., Radu C. (2021). Radiotherapy regimens for rectal cancer: long-term outcomes and health-related quality of life in the Stockholm III trial. BJS Open.

[9] Verweij M.E., Franzen J., van Grevenstein W.M.U., Verkooijen H.M., Intven M.P.W. (2023). Timing of rectal cancer surgery after short-course radiotherapy: national database study [published online ahead of print, 2023 May 12]. Br J Surg.

[10] Garcia-Aguilar J., Patil S., Gollub M.J. (2022). Organ preservation in patients with rectal adenocarcinoma treated with total neoadjuvant therapy. J Clin Oncol.

[11] Garcia-Aguilar J., Chow O.S., Smith D.D. (2015). Effect of adding mFOLFOX6 after neoadjuvant chemoradiation in locally advanced rectal cancer: a multicentre, phase 2 trial. Lancet Oncol.

[12] Erlandsson J., Holm T., Pettersson D. (2017). Optimal fractionation of preoperative radiotherapy and timing to surgery for rectal cancer (Stockholm III): a multicentre, randomised, non-blinded, phase 3, non-inferiority trial. Lancet Oncol.

[13] Verweij M.E., Hoendervangers S., von Hebel C.M. (2023). Patient- and physician-reported radiation-induced toxicity of short-course radiotherapy with a prolonged interval to surgery for rectal cancer. Colorectal Dis.

[14] Raaymakers B.W., Lagendijk J.J., Overweg J. (2009). Integrating a 1.5 T MRI scanner with a 6 MV accelerator: proof of concept. Phys Med Biol.

[15] Lagendijk J.J., Raaymakers B.W., van Vulpen M. (2014). The magnetic resonance imaging-linac system. Semin Radiat Oncol.

[16] Intven M.P.W., de Mol van Otterloo S.R., Mook S. (2021). Online adaptive MR-guided radiotherapy for rectal cancer; feasibility of the workflow on a 1.5T MR-linac: clinical implementation and initial experience. Radiother Oncol.

[17] Ingle M., White I., Chick J. (2023). Understanding the benefit of magnetic resonance-guided adaptive radiotherapy in rectal cancer patients: a single-centre Study. Clin Oncol (r Coll Radiol).

[18] Devlin L., Grocutt L., Hunter B. (2022). The in-silico feasibility of dose escalated, hypofractionated radiotherapy for rectal cancer. Clin Transl Radiat Oncol..

[19] Eijkelenkamp H., Boekhoff M.R., Verweij M.E., Peters F.P., Meijer G.J., Intven M.P.W. (2021). Planning target volume margin assessment for online adaptive MR-guided dose-escalation in rectal cancer on a 1.5 T MR-Linac. Radiother Oncol.

[20] de Mol van Otterloo S.R., Christodouleas J.P., Blezer E.L.A. (2020). The MOMENTUM study: an international registry for the evidence-based introduction of MR-guided adaptive therapy. Front Oncol.

[21] von Elm E., Altman D.G., Egger M. (2014). The Strengthening the Reporting of Observational Studies in Epidemiology (STROBE) Statement: guidelines for reporting observational studies. Int J Surg.

[22] Aaronson N.K., Ahmedzai S., Bergman B. (1993). The European Organization for Research and Treatment of Cancer QLQ-C30: a quality-of-life instrument for use in international clinical trials in oncology. J Natl Cancer Inst.

[23] Stiggelbout A.M., Kunneman M., Baas-Thijssen M.C. (2016). The EORTC QLQ-CR29 quality of life questionnaire for colorectal cancer: validation of the Dutch version. Qual Life Res.

[24] Weiser M.R. (2018). AJCC 8th edition: colorectal cancer. Ann Surg Oncol.

[25] Musoro J.Z., Coens C., Sprangers M.A.G. (2023). Minimally important differences for interpreting EORTC QLQ-C30 change scores over time: A synthesis across 21 clinical trials involving nine different cancer types [published online ahead of print, 2023 May 7]. Eur J Cancer.

[26] Folkesson J., Birgisson H., Pahlman L., Cedermark B., Glimelius B., Gunnarsson U. (2005). Swedish Rectal Cancer Trial: long lasting benefits from radiotherapy on survival and local recurrence rate. J Clin Oncol.

[27] Pach R., Richter P., Sierzega M., Papp N., Szczepanik A. (2021). preoperative short-course radiotherapy and surgery versus surgery alone for patients with rectal cancer: a propensity score-matched analysis at 18-year follow-up. Biomedicines.

[28] Kammar P.S., Garach N.R., Masillamany S., de'Souza A., Ostwal V., Saklani A.P. (2022). Downstaging in advanced rectal cancers: a propensity-matched comparison between short-course radiotherapy followed by chemotherapy and long-course chemoradiotherapy. Dis Colon Rectum.

[29] Liao C.K., Kuo Y.T., Lin Y.C., Chern Y.J., Hsu Y.J., Yu Y.L. (2022). Neoadjuvant short-course radiotherapy followed by consolidation chemotherapy before surgery for treating locally advanced rectal cancer: a systematic review and meta-analysis. Curr Oncol.

[30] Chin R.I., Roy A., Pedersen K.S. (2022). Clinical complete response in patients with rectal adenocarcinoma treated with short-course radiation therapy and nonoperative management. Int J Radiat Oncol Biol Phys.

[31] Rooney M.K., De B., Corrigan K. (2023). Patient-reported bowel function and bowel-related quality of life after pelvic radiation for rectal adenocarcinoma: the impact of radiation fractionation and surgical resection. Clin Colorectal Cancer.

[32] Glynne-Jones R., Hall M., Nagtegaal I.D. (2020). The optimal timing for the interval to surgery after short course preoperative radiotherapy (5 ×5 Gy) in rectal cancer - are we too eager for surgery?. Cancer Treat Rev.

[33] Pach R., Sierzega M., Szczepanik A., Popiela T., Richter P. (2021). Preoperative radiotherapy 5 × 5 Gy and short versus long interval between surgery for resectable rectal cancer: 10-Year follow-up of the randomised controlled trial. Radiother Oncol.

[34] Col N.F., Haugen V. (2022). Shared decision-making and short-course radiotherapy for operable rectal adenocarcinoma: a patient's right to choose. J Wound Ostomy Continence Nurs.

[35] Nilsson P.J., Ahlberg M., Kordnejad S., Holm T., Martling A. (2021). Organ preservation following short-course radiotherapy for rectal cancer. BJS Open.

[36] Custers P.A., van der Sande M.E., Grotenhuis B.A. (2023). Long-term quality of life and functional outcome of patients with rectal cancer following a watch-and-wait approach. JAMA Surg.

[37] Gilbert A, Homer V, Brock K, et al. Quality-of-life outcomes in older patients with early-stage rectal cancer receiving organ-preserving treatment with hypofractionated short-course radiotherapy followed by transanal endoscopic microsurgery (TREC): non-randomised registry of patients unsuitable for total mesorectal excision [published correction appears in Lancet Healthy Longev. 2022, 3(12):e816]. *Lancet Healthy Longev*. 2022;3(12):e825-e838. 10.1016/S2666-7568(22)00239-2. 10.1016/S2666-7568(22)00239-2 PMC9722406 36403589

[38] Bach S.P., Gilbert A., Brock K. (2021). Radical surgery versus organ preservation via short-course radiotherapy followed by transanal endoscopic microsurgery for early-stage rectal cancer (TREC): a randomised, open-label feasibility study. Lancet Gastroenterol Hepatol.

[39] Bonomo P., Lo Russo M., Nachbar M. (2020). 1.5 T MR-linac planning study to compare two different strategies of rectal boost irradiation. Clin Transl Radiat Oncol..

[40] Couwenberg A.M., Burbach J.P.M., Berbee M. (2020). Efficacy of dose-escalated chemoradiation on complete tumor response in patients with locally advanced rectal cancer (RECTAL-BOOST): a phase 2 randomized controlled trial. Int J Radiat Oncol Biol Phys.

[41] Verweij M.E., Hoendervangers S., Couwenberg A.M. (2022). Impact of dose-escalated chemoradiation on quality of life in patients with locally advanced rectal cancer: 2-year follow-up of the randomized RECTAL-BOOST Trial. Int J Radiat Oncol Biol Phys.

[42] Verweij M.E., Tanaka M.D., Kensen C.M. (2023). Towards Response ADAptive Radiotherapy for organ preservation for intermediate-risk rectal cancer (preRADAR): protocol of a phase I dose-escalation trial. BMJ Open..

