# Supplementary material for: Quality of life and clinical outcomes in rectal cancer patients treated on a 1.5T MR-Linac within the MOMENTUM study
Source: Clin Transl Radiat Oncol. 2024 Jan 4;45:100721. doi: 10.1016/j.ctro.2023.100721 (PMC10808928; doi:10.1016/j.ctro.2023.100721)
Supplement: Supplementary data 1 [file mmc1.docx]

**Appendix I.** Patient reported outcomes on relevant quality of life domains of the EORTC QLQ-C30 questionnaire in M0 patients without tumor resection or other additional therapy after radiotherapy at baseline and three, six and 12 months follow-up

|  | **Baseline (N=96)** | **3 months (N=42)** | **6 months (N=23)** | **12 months (N=16)** |
| --- | --- | --- | --- | --- |
| **EORTC QLQ-C30 completed** | 75 (78%) | 31 (74%) | 16 (70%) | 10 (62%) |
| **Global health score** |  |  |  |  |
| Mean (SD) | 77 (±17) | 76 (±16) | 77 (±16) | 86 (±11) |
| Missing | 21 (21.9%) | 11 (26.2%) | 7 (30.4%) | 6 (37.5%) |
| **Physical functioning** |  |  |  |  |
| Mean (SD) | 94 (±11) | 83 (±18) | 87 (±17) | 95 (±8.9) |
| Missing | 21 (21.9%) | 11 (26.2%) | 7 (30.4%) | 6 (37.5%) |
| **Role functioning** |  |  |  |  |
| Mean (SD) | 87 (±19) | 75 (±29) | 81 (±23) | 92 (±18) |
| Missing | 21 (21.9%) | 11 (26.2%) | 7 (30.4%) | 6 (37.5%) |
| **Emotional functioning** |  |  |  |  |
| Mean (SD) | 80 (±16) | 87 (±14) | 94 (±8.9) | 94 (±12) |
| Missing | 21 (21.9%) | 11 (26.2%) | 7 (30.4%) | 6 (37.5%) |
| **Cognitive functioning** |  |  |  |  |
| Mean (SD) | 92 (±14) | 90 (±13) | 88 (±20) | 97 (±7.0) |
| Missing | 22 (22.9%) | 11 (26.2%) | 7 (30.4%) | 6 (37.5%) |
| **Social functioning** |  |  |  |  |
| Mean (SD) | 91 (±15) | 82 (±21) | 85 (±20) | 93 (±14) |
| Missing | 21 (21.9%) | 11 (26.2%) | 7 (30.4%) | 6 (37.5%) |
| **Fatigue** |  |  |  |  |
| Mean (SD) | 17 (±18) | 27 (±24) | 19 (±18) | 10 (±14) |
| Missing | 21 (21.9%) | 11 (26.2%) | 7 (30.4%) | 6 (37.5%) |
| **Nausea/vomiting** |  |  |  |  |
| Mean (SD) | 2.7 (±7.3) | 6.5 (±12) | 3.1 (±6.7) | 0 (±0) |
| Missing | 21 (21.9%) | 11 (26.2%) | 7 (30.4%) | 7 (43.8%) |
| **Pain** |  |  |  |  |
| Mean (SD) | 8.6 (±15) | 14 (±22) | 15 (±16) | 1.7 (±5.3) |
| Missing | 22 (22.9%) | 12 (28.6%) | 7 (30.4%) | 6 (37.5%) |
| **Dyspnea** |  |  |  |  |
| Mean (SD) | 4.0 (±12) | 6.5 (±13) | 8.3 (±15) | 0 (±0) |
| Missing | 21 (21.9%) | 11 (26.2%) | 7 (30.4%) | 6 (37.5%) |
| **Insomnia** |  |  |  |  |
| Mean (SD) | 17 (±23) | 24 (±29) | 15 (±21) | 10 (±16) |
| Missing | 21 (21.9%) | 11 (26.2%) | 7 (30.4%) | 6 (37.5%) |
| **Loss of appetite** |  |  |  |  |
| Mean (SD) | 4.9 (±13) | 9.7 (±21) | 6.3 (±13) | 3.3 (±11) |
| Missing | 21 (21.9%) | 11 (26.2%) | 7 (30.4%) | 6 (37.5%) |
| **Constipation** |  |  |  |  |
| Mean (SD) | 11 (±21) | 6.5 (±16) | 2.1 (±8.3) | 0 (±0) |
| Missing | 21 (21.9%) | 11 (26.2%) | 7 (30.4%) | 6 (37.5%) |
| **Diarrhea** |  |  |  |  |
| Mean (SD) | 29 (±29) | 18 (±26) | 13 (±21) | 6.7 (±14) |
| Missing | 21 (21.9%) | 11 (26.2%) | 7 (30.4%) | 6 (37.5%) |

**Appendix II.** Patient reported outcomes on relevant quality of life domains of the EORTC QLQ-CR29 questionnaire in M0 patients without tumor resection or other additional therapy after radiotherapy at baseline and three, six and 12 months follow-up

|  | **Baseline (N=96)** | **3 months (N=42)** | **6 months (N=23)** | **12 months (N=16)** |
| --- | --- | --- | --- | --- |
| **EORTC QLQ-CR29 completed** | 68 (71%) | 30 (71%) | 16 (70%) | 10 (62%) |
| **Urinary frequency** |  |  |  |  |
| Mean (SD) | 27 (±23) | 28 (±23) | 28 (±26) | 17 (±31) |
| Missing | 28 (29.2%) | 12 (28.6%) | 8 (34.8%) | 6 (37.5%) |
| **Blood and mucus in stool** |  |  |  |  |
| Mean (SD) | 34 (±26) | 8.9 (±18) | 8.3 (±11) | 3.3 (±11) |
| Missing | 28 (29.2%) | 12 (28.6%) | 7 (30.4%) | 6 (37.5%) |
| **Stool frequency** |  |  |  |  |
| Mean (SD) | 28 (±24) | 20 (±24) | 22 (±27) | 17 (±31) |
| Missing | 29 (30.2%) | 13 (31.0%) | 7 (30.4%) | 6 (37.5%) |
| **Body image** |  |  |  |  |
| Mean (SD) | 10 (±17) | 6.7 (±12) | 13 (±23) | 5.6 (±12) |
| Missing | 30 (31.3%) | 12 (28.6%) | 7 (30.4%) | 6 (37.5%) |
| **Urinary incontinence** |  |  |  |  |
| Mean (SD) | 4.9 (±16) | 4.4 (±12) | 6.3 (±13) | 6.7 (±14) |
| Missing | 28 (29.2%) | 12 (28.6%) | 7 (30.4%) | 6 (37.5%) |
| **Dysuria** |  |  |  |  |
| Mean (SD) | 1.5 (±6.9) | 3.3 (±10) | 4.2 (±11) | 0 (±0) |
| Missing | 28 (29.2%) | 12 (28.6%) | 7 (30.4%) | 6 (37.5%) |
| **Abdominal pain** |  |  |  |  |
| Mean (SD) | 16 (±20) | 20 (±30) | 13 (±17) | 6.7 (±14) |
| Missing | 28 (29.2%) | 12 (28.6%) | 7 (30.4%) | 6 (37.5%) |
| **Buttock pain** |  |  |  |  |
| Mean (SD) | 14 (±21) | 16 (±26) | 13 (±21) | 13 (±17) |
| Missing | 28 (29.2%) | 12 (28.6%) | 7 (30.4%) | 6 (37.5%) |
| **Bloating** |  |  |  |  |
| Mean (SD) | 21 (±24) | 22 (±27) | 15 (±21) | 13 (±17) |
| Missing | 28 (29.2%) | 12 (28.6%) | 7 (30.4%) | 6 (37.5%) |
| **Anxiety** |  |  |  |  |
| Mean (SD) | 41 (±24) | 24 (±18) | 21 (±17) | 23 (±22) |
| Missing | 29 (30.2%) | 13 (31.0%) | 7 (30.4%) | 6 (37.5%) |
| **Weight** |  |  |  |  |
| Mean (SD) | 12 (±19) | 7.8 (±17) | 4.2 (±11) | 0 (±0) |
| Missing | 29 (30.2%) | 12 (28.6%) | 7 (30.4%) | 6 (37.5%) |
| **Flatulence** |  |  |  |  |
| Mean (SD) | 32 (±29) | 32 (±30) | 27 (±22) | 27 (±34) |
| Missing | 29 (30.2%) | 13 (31.0%) | 7 (30.4%) | 6 (37.5%) |
| **Faecal incontinence** |  |  |  |  |
| Mean (SD) | 14 (±21) | 14 (±23) | 8.3 (±15) | 3.3 (±11) |
| Missing | 29 (30.2%) | 13 (31.0%) | 7 (30.4%) | 6 (37.5%) |
| **Sore skin** |  |  |  |  |
| Mean (SD) | 9.0 (±19) | 11 (±16) | 8.3 (±15) | 10 (±16) |
| Missing | 29 (30.2%) | 13 (31.0%) | 7 (30.4%) | 6 (37.5%) |
| **Embarrassment** |  |  |  |  |
| Mean (SD) | 20 (±26) | 18 (±26) | 15 (±24) | 10 (±22) |
| Missing | 29 (30.2%) | 13 (31.0%) | 7 (30.4%) | 6 (37.5%) |
| **Sexual interest (men)** | **N = 71** | **N = 29** | **N = 16** | **N = 11** |
| Mean (SD) | 62 (±27) | 75 (±18) | 78 (±22) | 62 (±36) |
| Missing | 24 (33.8%) | 8 (27.6%) | 4 (25.0%) | 4 (36.4%) |
| **Impotence (men)** |  |  |  |  |
| Mean (SD) | 13 (±25) | 16 (±27) | 17 (±18) | 19 (±26) |
| Missing | 32 (45.1%) | 12 (41.4%) | 6 (37.5%) | 4 (36.4%) |
| **Sexual interest (women)** | **N = 25** | **N = 13** | **N = 7** | **N = 5** |
| Mean (SD) | 80 (±17) | 75 (±24) | 67 (±0) | 100 (±NA) |
| Missing | 7 (28.0%) | 5 (38.5%) | 3 (42.9%) | 4 (80.0%) |
| **Dyspareunia (women)** |  |  |  |  |
| Mean (SD) | 3.7 (±11) | 13 (±18) | 17 (±24) | 33 (±NA) |
| Missing | 16 (64.0%) | 8 (61.5%) | 5 (71.4%) | 4 (80.0%) |

**Appendix III.** Sensitivity analysis: mixed model analysis of patient reported outcomes on relevant quality of life domains of the EORTC QLQ-C30 questionnaire in 151 patients with organ-preserving therapy (including Transanal Endoscopic Microsurgery and other additional therapies after radiation treatment) at baseline and three, six and 12 months follow-up

| **Variable** | **Baseline** | **3 months follow-up** | | | | **6 months follow-up** | | | | **12 months follow-up** | | | |
| --- | --- | --- | --- | --- | --- | --- | --- | --- | --- | --- | --- | --- | --- |
|  | **Mean** | **Mean** | **MD** | **LCI** | **UCI** | **Mean** | **MD** | **LCI** | **UCI** | **Mean** | **MD** | **LCI** | **UCI** |
| Global health score | 74.9 | 70.9 | -4 | -8.1 | 0 | 69.6 | -5.3 | -10.1 | -0.6 | 77.9 | 3 | -3.8 | 9.7 |
| Physical functioning | 93.2 | **84.5** | **-8.7** | **-11.7** | **-5.7** | **84.7** | **-8.6** | **-12.1** | **-5** | 89.7 | -3.6 | -8.5 | 1.4 |
| Role functioning | 84.3 | **71** | **-13.2** | **-18.9** | **-7.6** | **72.5** | **-11.8** | **-18.6** | **-5** | 83.2 | -1 | -10.4 | 8.3 |
| Emotional functioning | 78 | 79.4 | 1.4 | -2.3 | 5.1 | 81.5 | 3.6 | -0.8 | 7.9 | 85.9 | 7.9 | 1.9 | 13.9 |
| Cognitive functioning | 91.9 | 89.1 | -2.9 | -6.3 | 0.5 | 85.5 | -6.4 | -10.4 | -2.4 | 91.9 | 0 | -5.7 | 5.6 |
| Social functioning | 88.4 | **80.4** | **-8** | **-12** | **-4.1** | **79.9** | **-8.5** | **-13.2** | **-3.7** | 86.4 | -1.9 | -8.4 | 4.6 |
| Fatigue | 18.5 | **32** | **13.4** | **8.5** | **18.4** | **27.9** | **9.4** | **3.5** | **15.3** | 20.4 | 1.9 | -6.2 | 10 |
| Nausea/vomiting | 2.3 | 8.1 | 5.8 | 3.2 | 8.5 | 5.7 | 3.4 | 0.3 | 6.5 | 0.5 | -1.8 | -6.1 | 2.6 |
| Pain | 10.8 | 17.6 | 6.8 | 1.6 | 12 | 19.2 | 8.4 | 2.3 | 14.6 | 6.9 | -3.8 | -12.3 | 4.6 |
| Dyspnea | 4.7 | 11.1 | 6.4 | 2.9 | 9.9 | 10 | 5.3 | 1.2 | 9.5 | 3.6 | -1.1 | -6.8 | 4.7 |
| Insomnia | 17.8 | 24.2 | 6.4 | -0.1 | 13 | 24.5 | 6.6 | -1.2 | 14.5 | 20.4 | 2.6 | -8.2 | 13.4 |
| Loss of appetite | 6.8 | 14.6 | 7.9 | 3.2 | 12.6 | 11.5 | 4.8 | -0.9 | 10.4 | 5.5 | -1.3 | -9 | 6.5 |
| Constipation | 9.5 | 11.3 | 1.8 | -2.3 | 5.9 | 10.3 | 0.8 | -4.1 | 5.8 | 6.5 | -3 | -9.8 | 3.8 |
| Diarrhea | 26.1 | **15.4** | **-10.7** | **-16.4** | **-5** | **16.7** | **-9.4** | **-16.2** | **-2.6** | **14.1** | **-12** | **-21.4** | **-2.6** |
| *MD, mean difference; LCI, lower confidence interval; UCI, upper confidence interval*  *Bold outcomes reflect statistically significant (P < 0.05) & clinically meaningful differences according to Musoro et al^27^for available quality of life domains or according to a minimally important difference of ≥10 point change otherwise* | | | | | | | | | | | | | |

**Appendix IV.** Sensitivity analysis: mixed model analysis of patient reported outcomes on relevant quality of life domains of the EORTC QLQ-CR29 questionnaire in 151 patients with organ-preserving therapy (including Transanal Endoscopic Microsurgery and other additional therapies after radiation treatment) at baseline and three, six and 12 months follow-up

| **Variable** | **Baseline** | **3 months follow-up** | | | | **6 months follow-up** | | | | **12 months follow-up** | | | |
| --- | --- | --- | --- | --- | --- | --- | --- | --- | --- | --- | --- | --- | --- |
|  | **Mean** | **Mean** | **MD** | **LCI** | **UCI** | **Mean** | **MD** | **LCI** | **UCI** | **Mean** | **MD** | **LCI** | **UCI** |
| Urinary frequency | 27.2 | 30 | 2.8 | -2.5 | 8.1 | 26 | -1.2 | -7.7 | 5.4 | 23.2 | -4 | -12.4 | 4.4 |
| Blood and mucus in stool | 30.9 | **9.8** | **-21.1** | **-26.7** | **-15.5** | **10.3** | **-20.6** | **-27.5** | **-13.8** | **10.1** | **-20.8** | **-29.8** | **-11.9** |
| Stool frequency | 27 | 21.1 | -5.8 | -12.1 | 0.4 | 20.4 | -6.5 | -14.1 | 1.1 | 23.5 | -3.4 | -13.2 | 6.4 |
| Body image | 11.8 | 11.9 | 0.1 | -3.8 | 4 | 14.7 | 2.9 | -1.9 | 7.7 | 15.5 | 3.7 | -2.4 | 9.9 |
| Urinary incontinence | 3.9 | 5.2 | 1.3 | -1.9 | 4.4 | 6.9 | 2.9 | -0.9 | 6.7 | 7.8 | 3.9 | -1 | 8.9 |
| Dysuria | 1.8 | 2.5 | 0.7 | -1.8 | 3.2 | 3.3 | 1.5 | -1.5 | 4.5 | 0 | -1.8 | -5.7 | 2.1 |
| Abdominal pain | 13.4 | 19 | 5.6 | 0.1 | 11.1 | 13.3 | -0.1 | -6.7 | 6.6 | 9.2 | -4.3 | -13 | 4.5 |
| Anal dysfunction | 17.9 | 19.9 | 2 | -4.1 | 8.2 | 21.9 | 4.1 | -3.4 | 11.5 | 15.6 | -2.2 | -12 | 7.5 |
| Bloating | 18.9 | 20 | 1.1 | -4 | 6.2 | 18.1 | -0.8 | -6.9 | 5.4 | 17.2 | -1.7 | -9.8 | 6.3 |
| Anxiety | 42 | 33.2 | -8.8 | -13.8 | -3.9 | **29.3** | **-12.7** | **-18.7** | **-6.7** | **30.5** | **-11.5** | **-19.3** | **-3.8** |
| Weight | 14.5 | 8.6 | -5.8 | -10.6 | -1.1 | 8.8 | -5.7 | -11.4 | 0.1 | 7.7 | -6.8 | -14.3 | 0.7 |
| Flatulence | 30 | 30.5 | 0.5 | -6.4 | 7.4 | 26.2 | -3.8 | -12.1 | 4.5 | 31 | 1 | -9.8 | 11.9 |
| Faecal incontinence | 14.4 | 10.6 | -3.8 | -8.8 | 1.1 | 12.3 | -2.1 | -8.1 | 3.9 | 12.2 | -2.2 | -9.9 | 5.5 |
| Sore skin | 12 | 11.8 | -0.3 | -5.1 | 4.6 | 13.5 | 1.5 | -4.5 | 7.4 | 14.6 | 2.6 | -5 | 10.2 |
| Embarrassment | 21.3 | 17 | -4.3 | -10.5 | 1.9 | 20.1 | -1.2 | -8.7 | 6.3 | 25.6 | 4.4 | -5.3 | 14 |
| Sexual interest (man) | 63.1 | 70.9 | 7.8 | 1.4 | 14.2 | 70.8 | 7.7 | 0.1 | 15.3 | 70 | 7 | -2.8 | 16.8 |
| Impotence | 13.9 | 16.1 | 2.2 | -4.9 | 9.3 | **24** | **10.1** | **2** | **18.2** | **32.9** | **19** | **9** | **28.9** |
| Sexual interest (woman) | 82.4 | 83.1 | 0.7 | -9 | 10.4 | 79.1 | -3.3 | -15.5 | 8.9 | 95.1 | 12.7 | -10.4 | 35.9 |
| Dyspareunia | 4.6 | 11.7 | 7.1 | -7.7 | 21.9 | 8.9 | 4.3 | -13.3 | 21.9 | 37.9 | 33.3 | 0 | 66.7 |
| *MD, mean difference; LCI, lower confidence interval; UCI, upper confidence interval*  *Bold outcomes reflect statistically significant (P < 0.05) & clinically meaningful differences according to a minimally important difference of ≥10 point change* | | | | | | | | | | | | | |

**Appendix V.** Clinical tumor response assessed on follow-up MRI and presence of recurrence in non-resected and resected patients, respectively, at three, six and 12 months of follow-up, stratified by disease stage at baseline

| **Disease stage** | **Low**  **(n = 9)** | | | **Intermediate**  **(n = 109)** | | | **LARC**  **(n = 36)** | | | **Metastatic**  **(n = 18)** | | | **Total**  **(n = 172)** | | |
| --- | --- | --- | --- | --- | --- | --- | --- | --- | --- | --- | --- | --- | --- | --- | --- |
| **Follow-up in months** | **3M** | **6M** | **12M** | **3M** | **6M** | **12M** | **3M** | **6M** | **12M** | **3M** | **6M** | **12M** | **3M** | **6M** | **12M** |
| **Number of patients** | **9** | **9** | **6** | **106** | **103** | **87** | **36** | **35** | **27** | **18** | **16** | **13** | **169** | **163** | **133** |
| **Non-resected, n (%)** | **9 (100)** | **4 (44)** | **1 (17)** | **37 (35)** | **13 (13)** | **9 (10)** | **31 (86)** | **16 (46)** | **7 (26)** | **14 (78)** | **8 (50)** | **-** | **91 (54)** | **41 (25)** | **17 (13)** |
| *Clinical response assessed, n (%)* | *9 (100)* | *3 (75)* | *1 (100)* | *31 (84)* | *12 (92)* | *9 (100)* | *13 (42)* | *15 (94)* | *6 (86)* | *11 (79)* | *7 (88)* | *-* | *64 (70)* | *37 (90)* | *16 (94)* |
| Complete response | 3 (33) | 3 (100) | 1 (100) | 4 (13) | 7 (58) | 7 (78) | 1  (8) | 4 (27) | 5 (83) | - | - | - | 8  (13) | 14 (38) | 13 (81) |
| Partial response | 6 (67) | - | - | 21 (68) | 4 (33) | 1 (11) | 10 (77) | 10 (67) | - | 7 (64) | 4 (57) | - | 44 (69) | 18 (49) | 1  (6) |
| Stable disease | - | - | - | 5 (16) | 1  (8) | 1 (11) | 1  (8) | 1  (7) | - | 1  (9) | 2 (29) | - | 7  (11) | 4 (11) | 1  (6) |
| Local progression | - | - | - | - | - | - | - | - | - | 1  (9) | - | - | 1  (2) | - | - |
| Distant progression | - | - | - | 1  (3) | - | - | - | - | 1 (17) | 1  (9) | 1 (14) | - | 2  (3) | 1 (3) | 1 (6) |
| Local & distant progression | - | - | - | - | - | - | 1 (8) | - | - | 1  (9) | - | - | 2  (3) | - | - |
| *Missing* | *-* | *1 (25)* | *-* | *6 (16)* | *1  (8)* | *-* | *18 (58)* | *1  (6)* | *1 (14)* | *3 (21)* | *1 (12)* | *-* | *27 (30)* | *6 (15)* | *1  (6)* |
| **Resected, n (%)** | **-** | **4 (44)** | **5 (83)** | **62 (58)** | **82 (80)** | **73 (84)** | **3  (8)** | **9 (26)** | **13 (48)** | **4 (22)** | **7 (44)** | **11 (85)** | **69 (41)** | **102 (63)** | **102 (77)** |
| *Disease recurrence assessed, n (%)* | *-* | *4 (100)* | *4 (80)* | *54 (87)* | *45 (55)* | *32 (44)* | *2 (67)* | *6 (67)* | *8 (62)* | *4 (100)* | *5 (71)* | *8 (73)* | *60 (87)* | *60 (59)* | *52 (51)* |
| No recurrence | - | 4 (100) | 4 (100) | 51 (94) | 40 (89) | 22 (69) | 2 (100) | 6 (100) | 8 (100) | 2 (50) | 2 (40) | 6 (75) | 55 (92) | 52 (87) | 40 (77) |
| Local recurrence | - | - | - | - | - | 1  (3) | - | - | - | 1 (25) | - | - | 1  (2) | - | 1  (2) |
| Distant recurrence | - | - | - | 3  (6) | 5  (11) | 9 (28) | - | - | - | 1 (25) | 2 (40) | 2 (25) | 4  (7) | 7  (12) | 11 (21) |
| Local & distant recurrence | - | - | - | - | - | - | - | - | - | - | 1 (20) | - | - | 1  (2) | - |
| *Missing* | *-* | *-* | *1 (20)* | *8 (13)* | *37 (45)* | *41 (56)* | *1 (33)* | *3 (33)* | *5 (38)* | *-* | *2 (29)* | *3 (27)* | *9 (13)* | *42 (41)* | *50 (49)* |
| **Resection unknown** | **-** | **1 (11)** | **-** | **7  (7)** | **8  (8)** | **5  (6)** | **2  (6)** | **10 (29)** | **7 (26)** | **-** | **1  (6)** | **2 (15)** | **9  (5)** | **20 (12)** | **14 (11)** |
| Clinical tumor response on MRI was assessed in patients without surgery; disease recurrence was assessed in patients who underwent tumor resection. Response and recurrence rates were calculated in patients in whom response and recurrence was assessed, respectively. | | | | | | | | | | | | | | | |
